# Blood Donation: Survey Results

**DOI:** 10.5505/tjh.2012.49091

**Published:** 2012-03-05

**Authors:** Gürsel Ersan, Fatma Liv, Şükran Köse

**Affiliations:** 1 İzmir Tepecik Education and Research Hospital, Blood Center, İzmir, Turkey; 2 İzmir Tepecik Education and Research Hospital, Department of Infectious Diseases and Clinical Microbiology, İzmir, Turkey

## TO THE EDITOR

A survey of 1735 blood centers (131) and hospitals (1604) in the US showed that blood collection rate per thousand US population of donor age between 18 and 65 years decreased from 85.6 (total number of collections, 13.890) in 2004 compared to 88.0 (total number of collections, 14.259) in 2001 (2.7 % decrease). As blood utilization increases with the advent of more complex therapeutic interventions, blood collection centers are finding it difficult to maintain an adequate blood inventory because the availability of blood for transfusion is dependent on volunteer donors [1]. In the US approximately 40% of the adult population is eligible to donate blood, but just 5% about 5% do so [2]. Moreover, only 3.5% of the age-eligible population donates blood in Australia. Donation anxiety due to a previous blood donation experience might be a major predictor of a donor’s future intention not to donate blood. Providing easy access to donation sites, overcoming time demands (such as scheduling appoinments for donation hours) and feeling confident for the procedure, more flexible donation hours, and increasing the number of sites where donation take place have positively influenced blood donation intentions [3]. The present study aimed to determine blood donors’ perceptions of the blood donation experience, behavioural and affective attitudes toward donation, and demographic characteristics that may influence their intention to donate blood in the future.

A self-administered questionnaire was used to collect data on the level of education, level of knowledge about blood donation, affective, and psychological components on blood donation decision emotional status, assessments of the physical condition of the blood donation center, treatment of donors by the staff who provide the technical and administrative oversight of blood donation, and the adequacy of informations provided in the blood donation form, satisfaction with the pre-donation physical examination, intend to return to the same center for future donations preference of the center for future, and perceptions of blood donation from 2000 first-time and repeat blood donors. The survey results show that more males than females donated blood, which may have been due to a prevalence of iron deficiency anemia among Turkish females resulting in ineligibility for donation or to inadequecy of women socialization in our country to participate in blood donation organizations. The Table 1 shows the donors’ demographic characteristics, distribution according to level of education, and regular monthly income. Increasing awareness of the importance of blood donation via education increased the number of volunteer donors that were more educated in developed countries. In the present study altruism was commonly (75%) reported as the reason for donating blood, as previously reported [3]. The belief of acquiring merit in God’s sight rated secondly (17 %), and lastly self-health issues perceived as “Blood donation is good for my health” (14%) determined donation intentions [4,5,6,7,8,9].

We think that increasing the sites where blood donation take place and improving the accessibility to these centers, implementation of educational programs designed to increase the awareness of blood donation among target populations (e.g. women), coping with the concerns about discomfort and fear of the donation experience, and creating attractive incentives for future donation might positively influence the rate of blood donation.

## CONFLICT OF INTEREST STATEMENT

The authors of this paper have no conflicts of interest, including specific financial interests, relationships, and/ or affiliations relevant to the subject matter or materials included.

## Figures and Tables

**Table 1:**
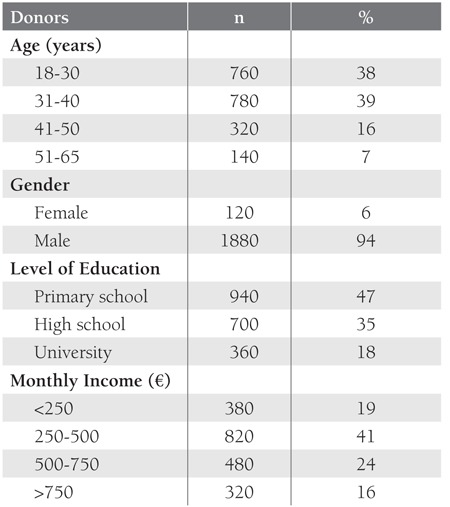
Donor Demographic Characteristics, Educational Status,and Monthly Income
